# Reward-Related Dorsal Striatal Activity Differences between Former and Current Cocaine Dependent Individuals during an Interactive Competitive Game

**DOI:** 10.1371/journal.pone.0034917

**Published:** 2012-05-14

**Authors:** Christopher J. Hyatt, Michal Assaf, Christine E. Muska, Rivkah I. Rosen, Andre D. Thomas, Matthew R. Johnson, Jennifer L. Hylton, Melissa M. Andrews, Brady A. Reynolds, John H. Krystal, Marc N. Potenza, Godfrey D. Pearlson

**Affiliations:** 1 Olin Neuropsychiatry Research Center, Institute of Living at Hartford Hospital, Hartford, Connecticut, United States of America; 2 Dept. of Psychiatry, Yale University School of Medicine, New Haven, Connecticut, United States of America; 3 Neurobiology, Yale University School of Medicine, New Haven, Connecticut, United States of America; 4 Child Study Center, Yale University School of Medicine, New Haven, Connecticut, United States of America; 5 Interdepartmental Neuroscience Program, Yale University, New Haven, Connecticut, United States of America; 6 Department of Pediatrics, The Ohio State University, Columbus, Ohio, United States of America; Bellvitge Biomedical Research Institute-IDIBELL, Spain

## Abstract

Cocaine addiction is characterized by impulsivity, impaired social relationships, and abnormal mesocorticolimbic reward processing, but their interrelationships relative to stages of cocaine addiction are unclear. We assessed blood-oxygenation-level dependent (BOLD) signal in ventral and dorsal striatum during functional magnetic resonance imaging (fMRI) in current (CCD; n = 30) and former (FCD; n = 28) cocaine dependent subjects as well as healthy control (HC; n = 31) subjects while playing an interactive competitive Domino game involving risk-taking and reward/punishment processing. Out-of-scanner impulsivity-related measures were also collected. Although both FCD and CCD subjects scored significantly higher on impulsivity-related measures than did HC subjects, only FCD subjects had differences in striatal activation, specifically showing hypoactivation during their response to gains versus losses in right dorsal caudate, a brain region linked to habituation, cocaine craving and addiction maintenance. Right caudate activity in FCD subjects also correlated negatively with impulsivity-related measures of self-reported compulsivity and sensitivity to reward. These findings suggest that remitted cocaine dependence is associated with striatal dysfunction during social reward processing in a manner linked to compulsivity and reward sensitivity measures. Future research should investigate the extent to which such differences might reflect underlying vulnerabilities linked to cocaine-using propensities (e.g., relapses).

## Introduction

Deficits in impulse control and reward processing are hypothesized to initiate and sustain cocaine dependence [1], [2], [3], [4], which is characterized by favoring immediate rewards of drug use over delayed non-drug rewards, despite potential negative consequences [5]. Mesocorticolimbic circuits, involving the dopaminergically innervated ventral and dorsal striatum as well as orbitofrontal and anterior cingulate cortices, are crucially involved in reward processing, and dysregulation in these circuits is implicated in both impulsivity and cocaine dependence [1], [6], [7], [8], [9], [10].

Abuse of substances, including cocaine [11] and alcohol [12], [13], [14], has been associated with reduced ventral striatal activity during non-drug reward anticipation or receipt, but the number of such studies is small and the origins of such hypoactivity are still poorly defined. One explanation for diminished mesocorticolimbic activation involves the reward-deficiency syndrome (RDS) hypothesis [15], which conjectures that drugs of abuse, due to their potent dopaminergic effects, normalize ventral striatal dopamine levels, whereas non-drug related rewards fail to do so, leading RDS individuals to seek cocaine or other abused drugs. Long-term, chronic cocaine abuse has been shown, however, to exacerbate underlying non-drug reward response deficiencies, through remodeling of neural circuitry [6], [16], [17], [18]. This so-called ‘hijacking’ of the reward system leads abusers to attribute even greater value to drug-related rewards at the expense of non-drug rewards [19], [20].

Two recent studies found, however, in apparent contradiction to the RDS hypothesis, greater ventral striatal activity in cocaine-dependent [21] or substance-dependent [22] individuals, when compared with healthy subjects, during non-drug reward anticipation or receipt. These studies lend support to the alternative ‘impulsivity hypothesis’ [23], related to opponent process theory, and which contrasts with the RDS hypothesis in that it predicts greater sensitivity even to non-drug rewards, along with an insensitivity to punishments [22], [24], [25]. Such discrepancies in recent studies of substance abuse and neural response to rewards clearly indicate that more research is needed to elucidate the effects of cocaine-abuse on the reward system, and the ventral striatum in particular, during non-drug reward anticipation and receipt.

As drug use and other addictive behaviors become habitual, striatal involvement may shift from ventral to dorsal [5], [26], [27], [28], [29]. Dorsal striatal-related networks are implicated in habitual behaviors [30], including cue-driven drug use and craving, and are theorized to contribute to compulsive cocaine use and relapse [26], [27], [28], [31], [32]. In support of this concept, two recent functional magnetic resonance imaging (fMRI) studies found that increased dorsal striatal activity was directly related to cocaine craving, either induced by psychological stress (in abstinent cocaine-dependent individuals in treatment) [33] or cocaine imagery (in actively abusing cocaine-dependent individuals) [5], [10]. Therefore, although cocaine use may be initiated by factors including trait impulsivity and supported by cocaine’s rewarding effects, habitual use in the later stages of addiction may depend less on the experience of cocaine reward and instead be both impulsively and compulsively driven, via ventral and dorsal striatum respectively, by cocaine cues that previously signaled reward [5], [6].

Impulsivity (e.g., delay discounting and impaired inhibition) and theoretically related constructs (e.g., compulsivity, risk-taking and sensation-seeking) can be quantified using self-report or laboratory-based measurements [34], [35]. Three recent studies demonstrated that impulsivity-related measures were associated with diminished ventral striatal activation during reward anticipation in individuals with pathological gambling [36], in detoxified alcohol-dependent subjects [12], and in nonalcoholic individuals who were family history positive (FHP) for alcoholism [14]. In the third study, impulsivity and related constructs were assessed using a principal-component-based factor analysis of five self-report and two laboratory-based impulsivity-related measures [37], which revealed that the multifaceted nature of impulsivity and related constructs was optimally described by five factors. One of the five factors loaded most strongly on two self-report measures (i.e., the Sensitivity to Punishment and Sensitivity to Reward Questionnaire and the Padua Inventory assessing compulsivity [38]) and was found to correlate negatively with ventral striatal activity. Such a factorial approach should provide a means to behaviorally quantify impulsivity domains in users of other addictive drugs as well, including cocaine, and explore correlates with brain function. However, a correlational analysis of a broad range of impulsivity-related measures with reward circuitry function in both former and current cocaine dependent individuals has not yet been reported.

Examining reward response in the context of risk-taking is important, as increased risk-taking behavior is commonly observed in individuals with the greatest likelihood to develop substance abuse or dependence [23]. The Domino fMRI task was originally introduced to examine the role of the amygdala in signaling prospective negative outcomes during risk-taking [39], [40], but has also been shown to strongly activate the reward system, particularly the ventral striatum, as a result of gains during gameplay [40], [41]. Therefore, in studies of substance-dependent individuals, the Domino task is uniquely suited to examine both dysfunctional brain reward processing and behavioral risk-taking, as well as their relationships with the five impulsivity-related constructs derived from the factor analysis described above.

Examination of reward system dysfunction in individuals in long-term cocaine abstinence, to our knowledge, has not yet been attempted. The inclusion of FCD individuals, who have been cocaine abstinent for at least six months, in addition to CCD individuals and healthy controls, is important because chronic cocaine use can induce changes in brain structure and function [27], [42]. Because the extent to which neural and behavioral recovery occurs with sustained abstinence is unclear, inclusion of FCD subjects permits direct comparisons with CCD individuals and might demonstrate in these subjects at least partial recovery of brain reward functioning to pre-morbid status.

Our goals in this study were as follows: 1) examine striatal activity, both ventral and dorsal, during reward receipt in both former and current cocaine-dependent individuals versus healthy controls, 2) assess risk-taking behavior in these same groups during Domino gameplay and 3) find relationships between neural reward activity and behavioral risk-taking with five impulsivity-related factors derived from a factor analysis study. In keeping with these goals, we hypothesized that both FCD and CCD subjects as compared with HC subjects would exhibit dysfunctional reward processing, assessed using fMRI during Domino gameplay. In particular, in accordance with the RDS hypothesis, we predicted that both ventral and dorsal striatum would show less activation in response to gains versus losses during gameplay in CCD and FCD compared with HC. We predicted, however, that FCD would exhibit less hypoactivity in these same brain regions than CCD, due to at least partial recovery of reward neurocircuitry to baseline. We also anticipated that both CCD and FCD groups, due to greater trait impulsivity, would exhibit greater risk-taking behavior during Domino gameplay than HC individuals, but the FCD group would exhibit less risk-taking than the CCD group, due to reduced impulsivity from cessation of chronic drug use. Lastly, we determined whether activity in these same striatal regions correlated with subjects’ impulsivity-related measures, hypothesizing that gain-related ventral striatal activation would correlate inversely with impulsivity-related factors across groups, based on findings in previous studies [12], [14], [36].

## Methods

### Ethics Statement

The study was approved by the Hartford Hospital and Yale University Institutional Review Boards and carried out at Hartford Hospital Institute of Living. All study participants provided written informed consent after the study had been fully explained to them. Participants were paid for participating in the imaging study.

### Study Participants

Participants included *n* = 36 CCD, *n* = 28 FCD and *n* = 33 HC subjects. After removing subjects for excessive reaction times (discussed later), 30 CCD, 28 FCD and 31 HC subjects remained. Table 1 (top) shows demographic data; groups were matched for age, gender and IQ. All subjects were right-handed. Participants were recruited by word-of-mouth, flyers, newspaper, online advertisement and drug abuse programs.

**Table 1:** Demographics, Drug Usage and Axis-I SCID data for HC, FCD and CCD groups.

Demographics	CCD (n = 30)			FCD (n = 28)			HC (n = 31)			ANOVA test	p-val
	Mean	SD	Range	Mean	SD	Range	Mean	SD	Range		
Age	37.9	8.1	22–55	37.9	8.1	21–50	35.6	7.4	25–59	*F*(2,86) = 0.887	*ns*
IQ	97.7	13.3	74–123	99.9	16.2	77–141	107.4	17.8	74–141	*F*(2,86) = 2.931	0.059
Gender (% M/F)	63.3/36.7			64.3/35.7			71.0/29.0			χ^2^(2) = 0.471	*ns*
Ethnicity (% W/B/H/A)	53.3/30.0/13.3/3.3			60.7/28.6/7.1/3.6			77.4/9.7/9.7/3.2			χ^2^(6) = 7.227	*ns*
**Cocaine Use**	**CCD (n = 30)**			**FCD (n = 28)**			**HC (n = 31)**			***t*** ** value^2^**	**p-val**
	**Mean**	**SD**	**Range**	**Mean**	**SD**	**Range**	**Mean**	**SD**	**Range**		
Age at first use (years)	20.5	6.3	13–42	*not available*			*N/A*			*N/A*	
Duration of use (years)	16.5	8.1	1.5–30	11.1	9.0	0.3–29	*N/A*			*t*(55) = 2.37	0.021
Amount used (weekly, USD)	$253	$317	$20–$1,400	$741	$911	$16–$2,632	*N/A*			*t*(49) = −2.65	0.011
Abstinence duration (years)	*N/A*			4.6	6.5	0.5–20	*N/A*			*N/A*	
Urine Test (pos/neg)^1^	20/10			0/28			0/31			*N/A*	
**Drug**	**Dependence/Abuse**			**Dependence/Abuse**			**Dependence/Abuse**				
	**C**	**P**		**C**	**P**		**C**	**P**			
Cocaine	27/3	N/A		0/0	25/3		0	0			
Alcohol	4/0	13/6		0/1	16/3		0	0			
Cannabis	4/3	10/6		1/1	10/2		0	0			
Opiate	9/0	1/1		0/0	7/1		0	0			
Amphetamine/Stimulant	0/0	0/1		0/0	2/1		0	0			
Hallucinogen/PCP	0/0	2/3		0/0	1/1		0	0			
Sedative/Anxiolytic	0/0	2/4		0/0	1/2		0	0			
Nicotine (daily smoker)	24	2		19	3		2	3			
**Other Axis-I**	**C**	**P**		**C**	**P**		**C**	**P**			
Major Depression	0	2		0	1		0	0			
Specific Phobia	1	0		1	0		0	0			
Panic Disorder	1	0		0	0		0	0			
Mood Disorder (SI)	0	2		0	5		0	0			
PTSD	0	1		0	4		0	0			
Dysthymic Disorder	0	0		1	0		0	0			
Bipolar I Disorder	0	0		0	1		0	0			

The DSM-IV diagnosis for current or past drug dependence or abuse is demarcated by a forward slash (e.g., 27/3 indicates 27 diagnosed with dependence and 3 with abuse of the given drug).

A, Asian; B, Black; F, female; H, Hispanic; M, male; *N/A*, not applicable; ns, non-significant; SD, standard deviation; W, white.

1 Day of scan only; ^2^two-sample *t*-test CCD versus FCD only.

C, Current; P, Past; USD, United States dollars; *N/A*, not applicable; PCP, phencyclidine; PTSD, post traumatic stress disorder; SI, substance induced.

All FCD and CCD subjects met DSM-IV criteria for dependence (for FCD, during previous use) based on initial screening. For inclusion into the study, FCD subjects were required to have ceased all cocaine use at least 6 months prior to the beginning of the study. Abstinence in FCD individuals was confirmed by self-report, urine toxicology screening, and where available, information from the substance abuse long-term follow-up groups that a proportion of subjects attended at the Institute of Living. For CCD subjects, participation required a positive cocaine urine test (last use <72 hours) on the day of screening. Frequency of self-reported use by CCD subjects in the 30 days prior to enrollment was as follows: <2 days: 3 subjects; 2–5 days: 6 subjects; 6–12 days: 9 subjects; 13 or more days: 10 subjects. Cocaine use data were not available for two CCD and one FCD subjects. Table 1 (*middle* and *bottom*) indicates substance abuse and Axis-I diagnoses, respectively.

Exclusion criteria for all subjects included: current non-substance Axis I disorders (e.g., schizophrenia), as assessed by structured clinical interview (SCID; [43]), current/past major neurological/physical illness, history of head trauma causing loss of consciousness, metallic objects in the body and estimated full-scale WAIS IQ <70. FCD were excluded for positive urine drug screen on the day of the scan and HC excluded for meeting DSM-IV criteria for substance abuse or dependence, except nicotine, or if their urine tested positive for recreational drugs on the day of testing. Nine CCD subjects had comorbid opiate use due to recruitment of cocaine dependent subjects from an outpatient drug abuse program (see Table 1). Cocaine use data were assessed using a self-report substance abuse questionnaire based on timeline follow-back methods [44]. Current and prior cocaine use data are in Table 1.

### Domino Task

**Figure 1:**
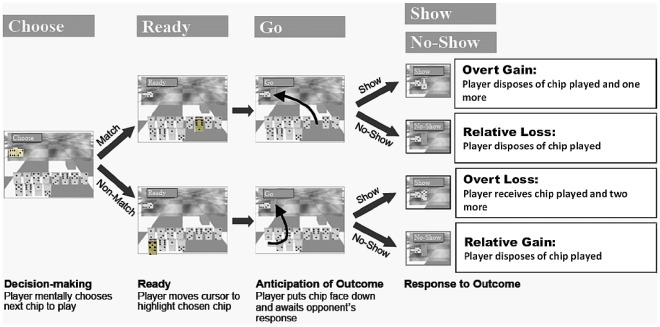
The Domino game task. The Domino game sequence and corresponding consequences are depicted. At the beginning of each round of the game the player must decide (mentally choose) what chip he/she will play next (i.e., ‘Choose’, the decision-making interval) and move the cursor to the selected chip when instructed (‘Ready’ interval). The chip can either match the opponent’s chip (*i.e.*, have one of the two numbers on the chip match one of those on the opponent’s chip, 6∶3 in this example; upper panel, 6∶1) or not (lower panel, 5∶2). After placing the selected chip face down next to the opponent’s chip, he/she awaits the opponent’s response (‘Go’ or ‘Anticipation of Outcome’ interval). The opponent can either challenge the player’s choice (‘show’) or not (‘no-show’). Based on the player’s choice and the opponent’s response, there are four possible consequences for each round during the ‘Response to Outcome’ interval: show match (*overt gain*); no-show match (*relative loss*, as the player could have been rewarded if challenged); show non-match (*overt loss*) and no-show non-match (*relative gain*, as the player successfully “bluffed”, that is, avoided punishment). The opponent’s chip and samples of matching and non-matching chips are highlighted (in yellow) for demonstration purposes only. In the actual scan, the game board and all chips are in color, not in grayscale as depicted in the figure. Also, all chips are the same size and color.

The Domino task is an event-related, two-player competitive computerized game modified from Kahn et al [39] described previously [41]. Figure 1 provides a graphical overview of the Domino task.

Participants practiced the game outside the scanner prior to scanning. Scanning began when the experimenter recognized that participants understood the game’s rules. A thorough debriefing was conducted immediately after scanning, where participants were asked about their emotions and strategies while playing. Open-ended questions and a Likert scale questionnaire were used where participants rated responses from 1 (least) to 5 (greatest) for agreement with statements (see example in “Results” section).

To characterize players’ game decisions, a Risk Index was defined as the ratio between the number of times a player chose a non-matching chip, only when a choice between non-matching and matching chips was available, to the total number of chips played (again, only when a choice between non-matching and matching chips was available).

The scanned subject is the player while a computer randomly generates the opponent’s responses. Subjects were told, however, that they were playing against a human opponent. Thus, from their perspective, subjects were playing in an interpersonal competitive context. Each game contains a pool of 28 domino-like game pieces. At the beginning of each game, 12 random domino chips are assigned to the player’s bank (shown face up on the computer screen), four undisclosed chips are randomly assigned to the opponent’s bank and a randomly chosen Master chip shows face-up on the board. The remaining 11 chips are not used. Each of the player’s chips is either a matching chip (has one of the two numbers on the Master chip) or a non-matching chip (has neither number on Master chip).

The player’s goal is to discard all assigned chips before the game ends (4 min) and, if they attain this goal, they are awarded $10, paid in cash at the end of the session. Thus, for the purposes of this study, during each round, discarding chips will be referred to as ‘gains’, while acquiring chips will be referred to as a ‘losses.’ Furthermore, as will be explained below, playing a matching chip is considered a ‘safe’ move, while playing a non-matching chip is considered a ‘risky’ move or ‘bluff’. It is only possible to win and to collect the resulting monetary bonus by occasionally ‘bluffing’ (i.e., playing a non-matching chip).

During each round of the game, the player selects a chip to play, places it face down adjacent to the Master chip and awaits the opponent’s response. The opponent can either challenge the player by asking him/her to reveal the chosen chip, or not challenge, allowing him/her to move on to the next round. Each round progresses according to the following commands, presented to the player both visually and aurally: (a) *Choose* instructs the subject to mentally select a chip to be played. The player can decide to pick either a matching or a non-matching chip. This is the ‘Decision-making’ interval; (b) *Ready* instructs the subject to move a cursor (using his/her dominant hand) to their chosen chip. This is the ‘Ready’ interval. These first two intervals each last 4 s; (c) *Go* instructs the player to press a button, as quickly as possible (note: the *Go* event duration is the subject’s reaction time (RT)), to put the chosen chip face down next to the Master chip.

The player then awaits the opponent’s response. This is the ‘Anticipation of Outcome’ interval with a ‘jittered’ duration of either 3.4, 5.4 or 7.4 s (5.4±2.0 s) [45]. The opponent’s response is either (d) *Show* or *No-Show*. The former command exposes the player’s selected chip, revealing whether they played ‘safe’ or ‘bluffed’, while the latter allows the player to discard his/her chip without exposing its value. This is the ‘Response to Outcome’ interval and its duration is also jittered (5.4±2.0 s). The next round of the game starts when the ‘Response to Outcome’ interval ends. The *Choose* command is then again presented to the player.

Based on the player’s choice and opponent’s response there are four possible consequences per round revealed during the ‘Response to Outcome’ interval: (1) Show Matching chip: a matching chip is exposed and the player is rewarded by discarding the selected chip plus one additional chip randomly chosen from his/her bank. This is an absolute gain. (2) Show Non-Matching chip: a non-matching chip is exposed, and the player is punished by acquiring the chip just played, plus two additional chips (from either the opponent’s bank or his/her previously discarded chips, thus not chosen by the player), for a total of three chips. This is an absolute loss; (3) No-Show of a Non-Match chip: a non-matching chip remains unexposed and then discarded, so the players successfully ‘bluff’, i.e., get away with a non-matching choice. This is a relative gain; and (4) No-Show of a Matching chip: a matching chip is not exposed and then discarded, so the player is relatively punished as he/she could have discarded another chip. This is a relative loss.

Rounds continue until the players win (by discarding all of their chips) or lose, when either 240 seconds have elapsed, or they have acquired all 16 available chips from the bank and the board. Participants played Domino games over two scan runs of 15 min each for a total of 8.39±0.61 games (Mean ± SD). Participants were told they were playing against the experimenter, whom they met prior to the scan, outside the scanner. The experimenter talked to the participant after each run making competitive comments about the games just played (such as “you really got me this time …”). To ensure that players were engaged in the game and believed that winning was possible, if they did not win during the first run, the first game of the second run was not automated and the experimenter “threw” the game, ensuring that the player won. Twenty-nine of the total of 89 players played a non-automated game (HC, 13 games; FCD, 6 games, CCD, 10 games); these games were excluded from the analysis. Games shorter than one minute (5.62% of all games) were also not analyzed.

### Behavioral Data Analysis

Likert scale score answers to the Domino debriefing statements were analyzed using non-parametric tests (Wilcoxon rank sum, Kruskal-Wallis) where appropriate, except for the case when determining if a given group response to a statement was statistically significantly greater than the middle score of 3. Here, a parametric one-sample *t*-test was used. SPSS™ software (v15, SPSS Inc., Chicago, IL) was used for all behavioral statistical analyses.

### Subject Exclusion

Subjects were excluded from analysis for mean reaction times (RTs) (during the *Go* event) in excess of 3.4 seconds, a value chosen based on the minimum stimulus duration of the ‘Anticipation of Outcome’ interval, is jittered (5.4±2.0 seconds). On the basis of excessive mean RTs, six CCD and two HC subjects were excluded.

### Impulsivity-related Measures

In a previous study that included many current study subjects [37], domains for multiple behavioral and self-report measures of impulsivity and related constructs were examined in At-Risk/Addiction subjects (individuals either family history positive for alcohol dependence, or current/former cocaine dependent) as well as healthy controls (14 CCD, 9 FCD and 21 HC from that study participated in the current study). Briefly, five widely-used, reliable and valid self-report questionnaires were used, and were described in that study: (i) the Behavioral Inhibition/Activation System (BIS/BAS) [46], (ii) the Barratt Impulsivity Scale (BIS-11) [47], (iii) the Sensitivity to Punishment and Sensitivity to Reward Questionnaire (SPSRQ) [48], (iv) the Sensation Seeking Scale (SSS Form V) [49] and (v) the Padua Inventory [50], plus two computer-based behavioral laboratory tests: (i) the Balloon Analog Risk Task (BART) [51] and (ii) the Experiential Discounting Task (EDT) [52].

In our current study, we used the previously identified Factor structure [37], i.e., we did not perform a new factor analysis, but rather used the five factor structure implicit in the *Component Score Coefficient* (CSC) *Matrix* calculated in the original Factor Analysis using Statistical Package for Social Sciences (SPSS), version 15.0 (SPSS Inc., Chicago, Illinois). Subjects taking the impulsivity-related test battery since the previous study, including subjects in this study, were added to the original database of *n* = 176 subjects and then new Z-scores for each impulsivity-related test were computed for all subjects (final *n* = 246). Updated Z-scores were multiplied by the CSC matrix to calculate new factor scores for our HC, FCD and CCD subjects.

### Functional MRI Acquisition

Blood oxygenation level dependent (BOLD) data were collected with a T2*-weighted echo planar imaging (EPI) sequence (TR/TE = 1860/27 msec, Flip angle = 70°, Field of view = 22 cm with a 64×64 acquisition matrix) using a Siemens Allegra 3 Tesla scanner. Thirty-six contiguous axial functional slices of 3 mm thickness with 1 mm gap were acquired, yielding 3.4×3.4×4.0 mm voxels. Overall, 492 images were acquired during each run, including six ‘dummy’ images at the beginning to allow global image intensity to reach equilibrium, which were excluded from data analysis.

### fMRI Data

#### Preprocessing

Imaging data were preprocessed using SPM2 (Wellcome Department of Cognitive Neurology, London, UK). Each individual’s data set was realigned to the first ‘non-dummy’ T2* image using the INRIAlign toolbox (A. Roche, EPIDAURE Group; http://www-sop.inria.fr/epidaure/software/INRIAlign) to compensate for any subject head movement. Movement parameters for each subject were then screened for excess head movement (>4 mm). The resulting images were spatially normalized to the Montreal Neurological Institute standard template [53] and spatially smoothed with a 9 mm isotropic (FWHM) Gaussian kernel. A high-pass filter with a cutoff of 128 s was applied to correct for EPI signal low-frequency drift.

### Events and Regressors

Functional MRI data were analyzed using a general linear model (GLM) approach using SPM5 (Wellcome Department of Cognitive Neurology, London, UK). As described previously [41], from the four game intervals described in detail in the online supplement, we created ten first-level (subject-level) regressors: (1) *choose-match* and *choose-nonmatch* from the ‘Decision-making’ interval; (2) *ready* from the ‘Ready’ interval; (3) *pick-match* and *pick-nonmatch* from the ‘Anticipation of Outcome’ interval; (4) *show-match*, *show-nonmatch*, *noshow-match* and *noshow-nonmatch* from the ‘Response to Outcome’ interval, each regressor corresponding to the four consequences; (5) and finally a *misc* regressor for events of non-interest including *Go* events and between-game events, during which the subject learned whether they won or lost the last game and then waited for the next game to being. The *misc* regressor also included all events occurring during games of less than one-minute duration. These events were not analyzed.

Regressors were modeled as boxcar functions convolved with the SPM5 canonical hemodynamic response function (HRF) and included HRF temporal derivatives. Regressors also included the six movement parameters (translation: *x*, *y* and *z* and rotation: pitch, roll and yaw).

In a fixed-effects first-level analysis, individual statistical parametric maps and contrast images were calculated and were composed of contrasts from the four ‘Response to Outcome’ interval regressors. These include the *Gain* contrast, a linear combination of the *show-match* and *noshow-nonmatch* regressors compared with an implicit baseline, the *Loss* contrast, a linear combination of the *show-nonmatch* and *noshow-match* regressors compared with an implicit baseline, and finally the *Gain-Loss* difference contrast.

### Statistical Analyses

We delineated the reward network using a random-effects second-level analysis one-sample *t*-test of the *Gain-Loss* contrast across all subjects (*n* = 89). We thresholded the resulting statistical parametric map at *p*<0.05 family-wise error (FWE) rate whole-brain corrected. We used the across-all-subjects *Gain-Loss* contrast map as an inclusive mask in all subsequent analyses, such that they examined group differences within the brain reward network only. We hereafter refer to this inclusive mask as the Reward mask, and the brain regions it defines as the Reward network.

To explore group differences in the *Gain-Loss* contrast we performed a random-effects repeated-measures between-subjects analysis of variance (ANOVA) in which we compared the three HC, FCD and CCD groups. We were specifically interested in between-group differences in *Gain-Loss* activation in targeted regions of interest (ROIs), ventral and dorsal striatum, according to our hypotheses. We first applied the Reward mask, as described previously, to the results from the one-way ANOVA between-group *Gain-Loss* contrast comparison. The statistical threshold for clusters occurring in ROIs was then set to *p*<0.05 uncorrected (due to an anticipated loss of power from a between-group ANOVA second-level analysis) with a minimum cluster size of *k* = 10 contiguous voxels (>270 mm^3^). The SPM5 toolbox ‘rfxplot’ was used to calculate effects sizes for the random effects group SPM5 analyses and was also used to create subsequent effect size bar graphs [54].

### Correlation of fMRI with Impulsivity-related Measures

To determine the relationship between impulsivity-related measures and reward-related brain activity, we performed a random-effects multiple regression analysis in SPM5 to obtain the correlation of each subject’s impulsivity-related factor score with the corresponding *Gain-Loss* contrast at each voxel. Only impulsivity-related factors (from the set of five factors described earlier) that were significantly different among the three groups were subject to correlation analysis.

As with the between-group ANOVA analysis, we first masked the multiple regression analysis using the Reward mask. We then conducted a between-group ANOVA on the multiple regression results to determine clusters with significant between-group differences for each impulsivity-related measure versus *Gain-Loss* contrast correlation. As with main-effect ANOVA described previously, we present only those between-group ANOVA correlation clusters occurring in ROIs, and set the statistical threshold to *p*<0.05 uncorrected with a minimum cluster size of *k* = 10 voxels.

*Post hoc* analyses were conducted to determine which group(s) differed in correlation between subjects’ impulsivity-related factor scores and corresponding *Gain-Loss* contrast. Statistical thresholds for *post-hoc* regression, performed on each group separately, were set at *q*<0.05 false-discovery rate (FDR) corrected (restricted to those voxels included in the Reward network) with a minimum cluster size of *k* = 10 contiguous voxels. Regression data for presentation for each group, as before, were further restricted to voxels in clusters located in ROIs. Data were obtained from the mean *Gain-Loss* contrast value of all voxels within a 5 mm radius of the peak voxel and were extracted using the eigenvariate option in SPM5.

## Results

### Behavioral Analyses

#### Game information

HC, FCD and CCD groups had 79.7±6.6, 82.8±4.0 and 80.6±5.7 ‘Response to Outcome’ events per subject, respectively, over the course of their two 15-minute scanning sessions (group differences, N.S., *F* = 2.363, *df* = 2,88, *P* = 0.100). The percentage of these events that were *Gain* events for HC, FCD and CCD were 50.9±6.1%, 48.8±6.0% and 48.7±5.9%, respectively, demonstrating that ‘Response to Outcome’ events for all three groups were divided approximately evenly between *Gain* and *Loss* events (group differences, N.S., *F* = 1.295, *df* = 2,88, *P* = 0.279). Not including the ‘thrown’ games (see online supplement), HC, FCD and CCD subjects won 20.2±11.5%, 25.6±12.8% and 22.8±12.6% of their games, respectively (group differences, N.S., *F* = 1.425, *df* = 2,88, *P* = 0.246).

#### Domino debriefing

Four statements on the Likert scale questionnaire were intended to reveal subject’s emotional reactions to the four possible outcomes of each move during the ‘Response to Outcome’ interval (see Table 2). Kruskal-Wallis tests indicated no differences among the three groups in mean responses to any statement. One-sample *t*-tests showed that, for all three groups, Likert scale responses to both absolute and relative gains (“I felt glad when …”) were statistically significant with regard to agreement with the statements (i.e., mean response >3) while responses to both absolute and relative losses (“I felt unhappy when …”) were not (see Table 2), suggesting that subjects were more motivated by gains than losses, consistent with previous results [41].

**Table 2:** Statistical group comparisons for responses to four attitude statements (AS).

AS1:	I felt glad when a matching chip was challenged (show match: **absolute gain**)									
AS2:	I felt glad when a non-matching was not challenged (no-show non-match: **relative gain**)									
AS3:	I felt unhappy when a non-matching chip was challenged (show non-match: **absolute loss**)									
AS4:	I felt unhappy when a matching chip was not challenged (no-show match: **relative loss**)									
***Responses by Group***										
*Group*	**AS1**		**AS2**			**AS3**		**AS4**		
	***absolute gain***	*P-value* ≤3†	***relative gain***		*P-value* ≤3†	***absolute loss***	*P-value* ≤3†	***relative loss***		*P-value* ≤3†
*hc (n = 31)*	3.97±1.02	<0.001	3.83±1.00		<0.001	3.07±1.10	= 0.369	2.93±1.33		= 0.391
*fcd (n = 28)*	4.00±1.05	<0.001	3.79±1.23		<0.001	3.22±1.28	= 0.188	3.29±1.27		= 0.123
*ccd (n = 30)*	4.21±1.07	<0.001	3.92±1.38		<0.001	3.39±1.45	= 0.081	3.18±1.42		= 0.255
***Kruskal-Wallis test for Group differences***										
	**AS1**			**AS2**		**AS3**			**AS4**	
χ^2^	1.665			0.874		1.157			0.955	
*P-value*	0.435			0.646		0.561			0.620	

From the Domino Debriefing Questionnaire (DDQ) regarding absolute and relative gains and losses during the ‘Response to Outcome’ interval. Responses are Likert scale for agreement with each statement: 1 = ’Not at all’ through 5 = ’Very much’. *Top*, Table values are the response mean and standard deviation for each group. *Bottom*, Kruskal-Wallis test results for group differences.

† = Null hypothesis, mean response ≤3.

For the HC, FCD and CCD groups, paired Wilcoxon signed-rank tests comparing subjects’ responses to statements regarding absolute versus relative gains (i.e., AS1 versus AS2), and absolute versus relative losses (i.e., AS3 versus AS4), showed no significant differences (AS1 vs. AS2, *P*>0.100 and AS3 vs. AS4, *P*>0.100, for all three groups; see Table 2 for AS definitions). Therefore, we concluded that subjects did not perceive absolute gain and loss events as more emotionally salient than the relative events. For this reason, in our fMRI GLM analyses, we grouped the relative and absolute gain event regressors together as the contrast ‘*Gain*’, and, likewise, we grouped relative and absolute losses event regressors together as the contrast ‘*Loss*’.

#### Risk behavior

A two-way ANOVA (3×4) of Risk-Index with the two factors Group (i.e., the three groups, HC, FCD and CCD) and Time (elapsed minutes into game, binned into four one minute intervals), respectively, revealed a significant main effect of Time (*F* = 13.802, *df* = 3,258, *P*<0.0001). Players tended to ‘bluff’ their opponent more towards the game end than beginning. There was no significant effect of Group (*F* = 0.723, *df* = 2,86, *P* = 0.488) and no significant interaction between Group and Time (*F* = 0.539, *df* = 6,258, *P* = 0.778).

#### Impulsivity-related constructs

A one-way ANOVA of the five factors described previously [37] involving all study subjects revealed significant group differences only for Factor 2, ‘*Self-Reported Compulsivity and Reward-Punishment Sensitivity*’ (*F* = 5.373, *df*  =  2,86, *P* = 0.030 (Bonferroni corrected)). Post-hoc analyses of Factor 2 revealed that HC scored lower than CCD subjects (Tukey’s HSD, *P* = 0.007) and trended towards scoring lower than FCD subjects (Tukey’s HSD, *P* = 0.054)).

#### Reaction times

For subjects not excluded from the study for excessively long RTs (>3.4 seconds), a one-way ANOVA analysis revealed no significant difference among groups for RTs (*F* = 2.805, *df* = 2,86, *P* = 0.066; HC: 877±775 ms, FCD: 1,090±921 ms and CCD: 1,291±1,020 ms).

### fMRI Analyses

We focused fMRI analyses exclusively on brain activity during the ‘Response to Outcome’ interval, both for the overall group analysis and for between-group comparisons, with the principal contrast of interest being *Gain-Loss*.

#### One-sample t-test: Across-all-subjects reward network

**Figure 2:**
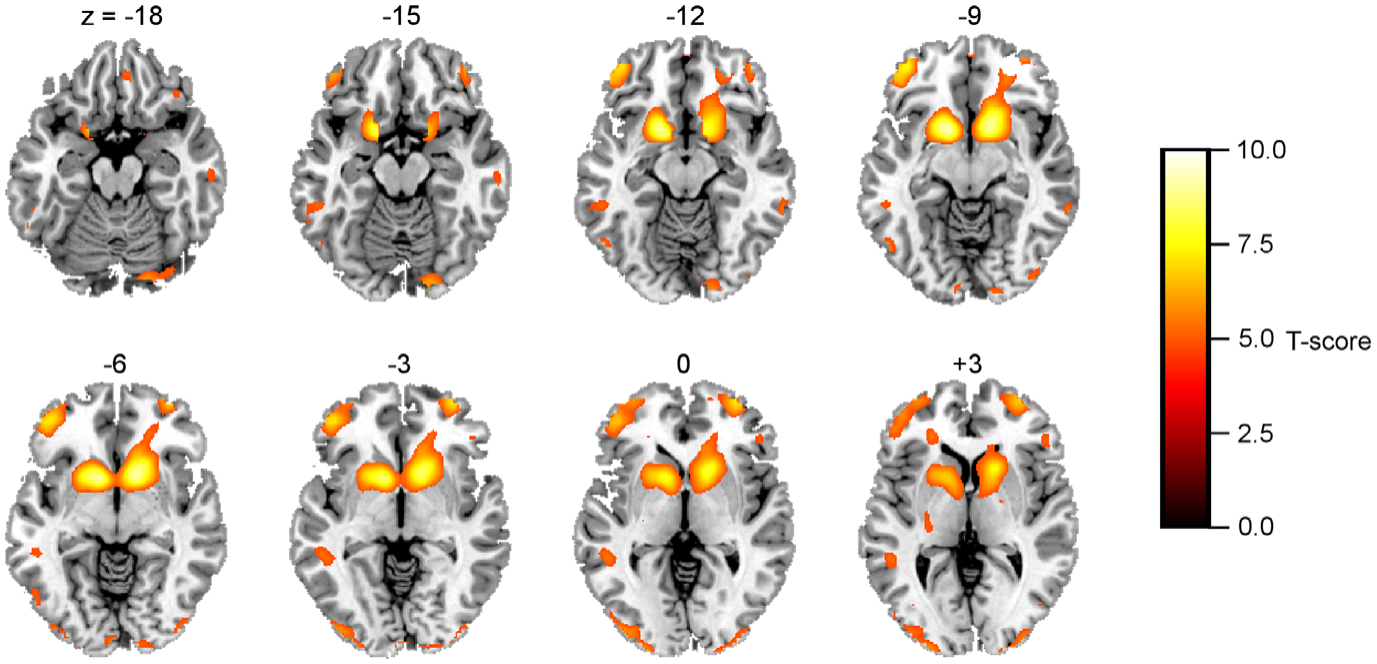
Statistical parametric one-sample *t*-maps of the *Gain-Loss* contrast for all groups combined (*n* = 89 subjects) that delineates the “Reward network”. Axial slices are labeled from z  = −18 mm to z  = +3 mm in steps of 3 mm. The threshold was set at *p*<0.05, FWE whole-brain corrected.

A one-sample *t*-test for the *Gain-Loss* contrast across all subjects from the three groups (n = 89), showed strong activity in reward-related brain regions including bilateral ventral striatum, right dorsal striatum (caudate) and left and right lateral orbitofrontal cortex (see Figure 2, *p*<0.05, FWE whole-brain corrected). Table 3, *top* provides the locations (MNI coordinates) and *t-*scores for each region of significant activity.

**Table 3:** Statistical parametric mapping results for the *Gain-Loss* contrast.

One-sample *t*-test: across-all-subjects, Reward network					
Anatomic location of maximum activation	MNI coordinates			*Gain-Loss*	
	x	y	Z	*T*-Score, *df* = 86	
L ventral striatum	−15	9	−9	9.32	
R ventral striatum	12	12	−6	9.20	
L OFC	−39	51	−9	7.46	
R OFC	33	60	−3	7.34	
L sup parietal lobe	−42	−36	39	6.86	
R sup parietal lobe	39	−36	39	6.54	
Threshold: *p*<0.05 FWE whole-brain corrected, minimum cluster size, *k* = 10 voxels					
OFC, orbitofrontal cortex; sup, superior					
**One-way ANOVA: Main Effect of Group, Reward network**					
**Anatomic location of maximum between-group difference in activation**	**MNI coordinates**			***Gain-Loss***	
	x	y	Z	***F*** **-score (** ***df*** ** = 2,86)**	***P*** ** (uncorr.)**
R dorsal caudate	18	18	9	5.16	0.0076
Threshold: *p*<0.05 uncorrected, minimum cluster size, *k* = 10 voxels; masked with Reward mask					

Across all groups one-sample *t*-test (*top*) and for the between-group ANOVA main effect, masked with the Reward mask (*bottom*).

#### One-way ANOVA: Main effect of group

**Figure 3:**
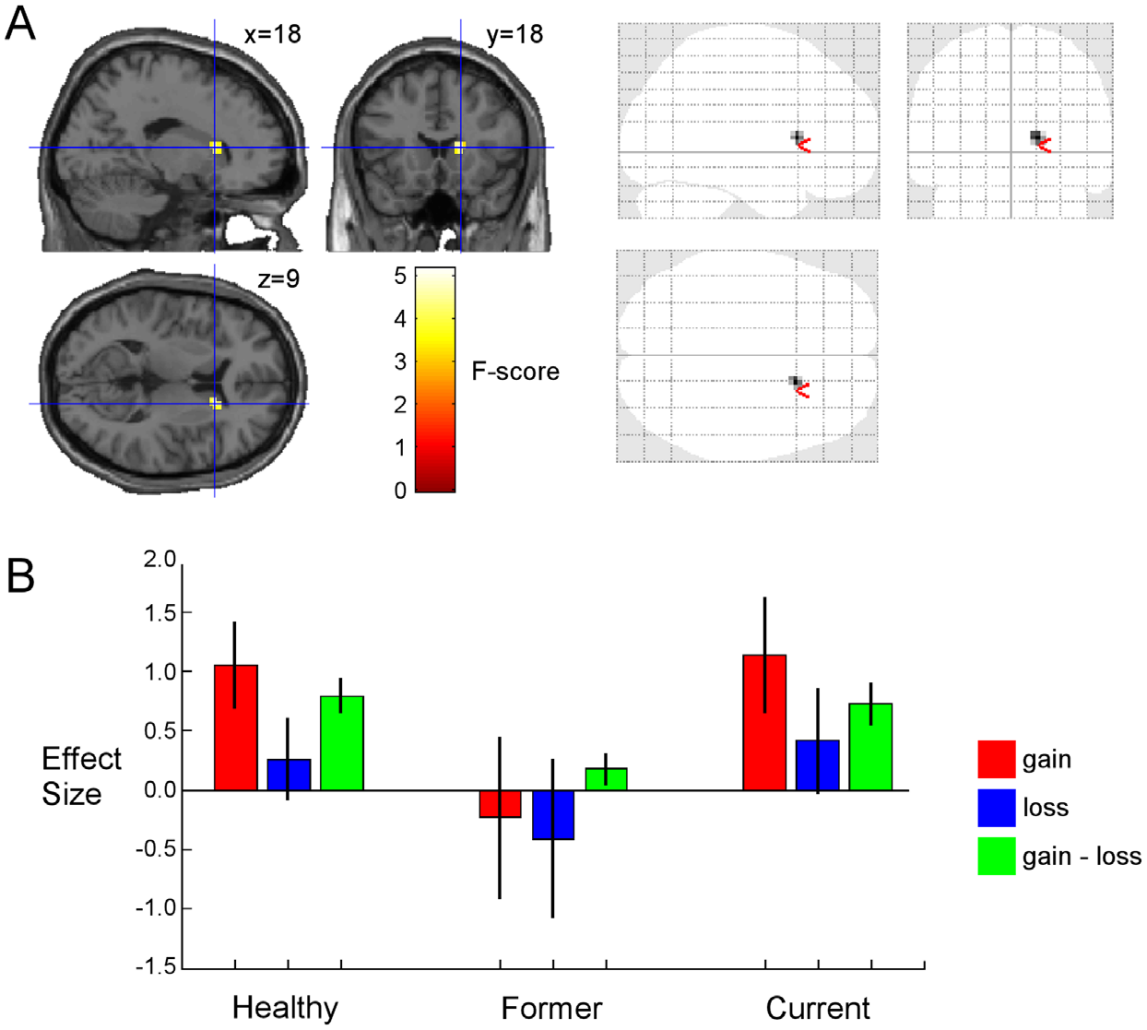
*A.* Statistical parametric *F*-maps (sagittal, coronal and axial) of the *Gain-Loss* contrast for one-way ANOVA between-group main effect (masked with the Reward mask). Crosshairs overlaid on brain slices are at located at *x,y,z*  = 18,18,9 (peak voxel). Glass brain at top right shows that the cluster in the right dorsal caudate is only surviving cluster. Threshold was set at *p*<0.05 uncorrected, minimum cluster size *k* = 10 voxels. *B.* Effect sizes for *Gain* (red), *Loss* (blue) and *Gain-Loss* (green) contrasts for HC, FCD and CCD groups at *x,y,z  = *18,18,9. Black bar represents standard error of the mean.

The masked one-way ANOVA analysis of the between-group differences for the *Gain-Loss* contrast yielded only one brain region with a group difference in activity, an ROI cluster (*k* = 18 voxels) in the right dorsal caudate (*p*<0.05 uncorrected, minimum cluster size *k* = 10; see Figure 3, panel A). Table 3, *bottom,* provides the location (MNI coordinates) and statistics for the right dorsal caudate region of significant between-group difference in activity.

Effect sizes for *Gain*, *Loss*, and for *Gain-Loss*, for each of the three groups at the peak main effect of Group activation in the right dorsal caudate are shown in Figure 3, panel B. *Gain-Loss* effect size is similar for both HC and CCD subjects, but is significantly decreased for FCD subjects (*F* = 4.764, *df* = 2,86, *p* = 0.011; post-hoc Tukey’s HSD: FCD < HC, *P* = 0.015; FCD < CCD, *p* = 0.037).

#### Impulsivity-related scores: Correlation analysis between-group ANOVA

**Figure 4:**
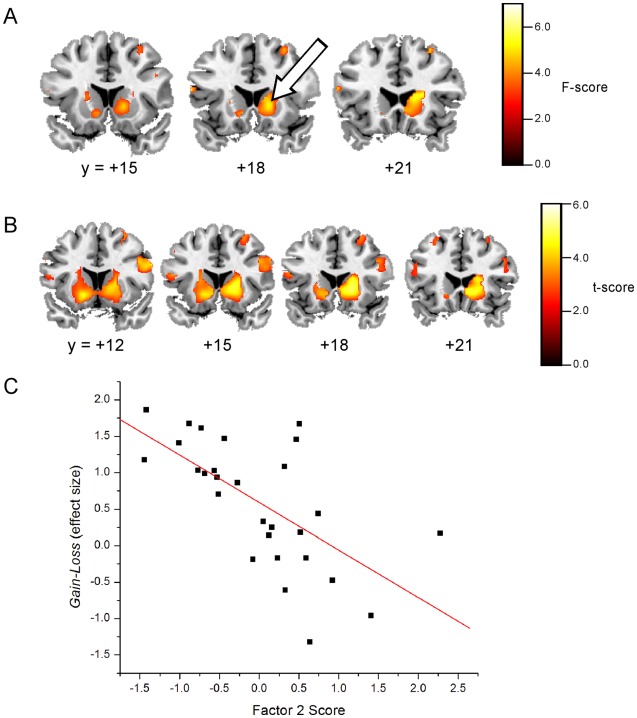
*A*. Statistical parametric *F*-maps (coronal slices; *y-*dimension shown) of the one-way between-groups ANOVA multiple regression analysis of Factor 2 scores versus *Gain-Loss* contrast. Block white arrow points to the right dorsal caudate cluster that overlaps with the right dorsal caudate cluster shown in Figure 4, panel A. *B*. Statistical parametric *t*-maps (coronal slices; *y-*dimension shown) for the *post hoc* FCD group multiple regression analysis of Factor 2 scores versus *Gain-Loss* contrast. Threshold was set at *q*<0.05 FDR corrected; minimum cluster size *k* = 10 voxels (masked with the Reward mask). *C*. Plot of the *Gain-Loss* effect size versus Factor 2 score regression analysis, with each *Gain-Loss* contrast value being the mean value in a 5 mm radius sphere centered at peak voxel *x,y,z  = *18,18,0 for each subject in the FCD group. The correlation coefficient of the fitted line was *R* = −0.641 (*p* = 0.0002 uncorrected).

A between-group ANOVA multiple regression analysis of Factor 2 impulsivity scores versus *Gain-Loss* contrast revealed several clusters within the Reward network at *p*<0.05 uncorrected, minimum cluster size, *k* = 10 voxels (see Table 4). One cluster was located in an ROI, the right dorsal caudate, as shown by the block arrow in Figure 4, panel A, at essentially the same location as the right dorsal caudate cluster in the between-group ANOVA main effect shown in Figure 3, panel A (peak cluster voxels at *x,y,z*  = 15,18,0 vs. 18,18,9).

**Table 4:** Between-group ANOVA multiple regression results: *Gain-Loss* contrast versus impulsivity-related Factor 2 scores.

Anatomic location of maximum between-group difference in correlation	MNI coordinates			Factor 2 vs *Gain-Loss* Correlation	
	x	y	z	*F*-score (*df* = 2,83)	*P* (uncorr.)
**Region of Interest clusters**					
R dorsal caudate	15	18	0	5.27	0.007
**Other Reward network clusters**					
R OFC	21	33	−9	7.96	0.001
L IFG, triangular	−51	39	6	7.10	0.001
R IFG, opercular	57	9	27	5.00	0.009
L middle frontal gyrus	−36	60	9	4.59	0.013
L precentral gyrus	−51	3	24	5.54	0.006
L precentral gyrus	−42	−3	57	4.69	0.012
L superior parietal lobe	−27	−57	66	7.82	0.001
R precuneus	6	−66	45	5.17	0.008

Threshold: *p*<0.05 uncorrected, minimum cluster size, *k* = 10 voxels; masked with Reward mask.

IFG, inferior frontal gyrus.

#### Impulsivity-related scores: Post-hoc correlation analysis

A post-hoc analysis of the between-group ANOVA multiple regression results indicated that FCD was significantly different than HC and CCD. Only FCD had surviving clusters within the Reward network at a threshold of *q*<0.05 FDR corrected (minimum cluster size *k* = 10 voxels). All correlation clusters within the Reward network for the FCD group, including those not located in ROIs, are described in Table 5. The ROI cluster in the right dorsal caudate (see Figure 4, panel B, coronal slice *y*  = +18 mm), in particular, overlaps the same right dorsal caudate ROI cluster found in the main effect of *Gain-Loss* (Figure 3, panel A). In this right dorsal caudate ROI cluster, FCD Factor 2 impulsivity scores were significantly negatively correlated with *Gain-Loss* effect size (*R*  = −0.641, *p* = 0.0002; 5 mm radius sphere centered at *x,y,z*  = 18,18,0) (see plot, Figure 4, panel C).

**Table 5:** FCD group *post-hoc* multiple regression results for *Gain-Loss* contrast versus impulsivity Factor 2 scores.

Anatomic location of maximum correlation	MNI coordinates			Factor 2 vs *Gain-Loss* Correlation		
	x	y	z	*t*-score (*df* = 26)	*P* (uncorr.)	*R*
**Region of Interest clusters**						
R dorsal caudate	18	18	0	4.29	0.0002	−0.644
L ventral striatum	−15	12	−6	3.89	0.0006	−0.607
R ventral striatum	15	12	−3	3.39	0.0022	−0.554
**Other Reward network clusters**						
R IFG, opercular	54	9	24	3.84	0.0007	−0.602
L superior parietal lobe	−24	−57	63	5.18	<0.0001	−0.712
L precentral gyrus	−33	−3	63	4.03	0.0004	−0.620
R precuneus	12	−72	57	3.57	0.0014	−0.574
R precentral gyrus	36	−9	66	4.24	0.0002	−0.639

Threshold: *q*<0.05 FDR correction, minimum cluster size, *k* = 10 voxels; masked with Reward mask.

#### Additional findings

No correlation was found between abstinence durations for FCD subjects with *Gain-Loss* effect size in any ROI. Additionally, we found no difference in HC, CCD and FCD subjects’ risk-taking behavior during gameplay as measured using the Risk Index.

#### Prior psychiatric disorders: Post hoc analysis

To determine if prior psychiatric disorders in the FCD and CCD groups had an impact on our findings, we repeated our analyses excluding the eight FCD and three CCD subjects who had prior psychiatric diagnoses of mood disorders or PTSD. There were, however, no qualitative differences between our original analysis and the post hoc analysis. The post hoc analysis still revealed relative hypoactivity for the FCD group in the right dorsal caudate (peak voxel at *x,y,z* = 18,18,9; post hoc vs. original: *F* = 3.73 (*df*  = 2,75) vs. *F* = 5.17 (*df* = 2,86); *p* = 0.029 vs. *p* = 0.008; cluster size *k* = 5 vs. *k* = 18), as well as an inverse correlation (*R* = −0.645, *df* = 18; *P* = 0.0021 vs. *R* = −0.641, *P* = 0.0002, *df* = 26) of FCD impulsivity-related Factor 2 scores with *Gain-Loss* activity in the right dorsal caudate. Quantitative differences can likely be attributed to reduced power in the post hoc analysis.

## Discussion

Our principal finding was that while engaged in the Domino task, involving risk-reward decision-making, FCD, CCD and HC subjects showed largely similar behavioral and brain responses. However, a neural between-group difference was found in the dorsal caudate, such that FCD subjects compared to HC and CCD showed reduced BOLD activation during response to gains. Interestingly, in the same region (i.e., right dorsal caudate), only FCD subjects’ *Gain-Loss* activity correlated inversely with their scores on impulsivity-related Factor 2, ‘Self-reported compulsivity and sensitivity to reward and punishments.’ Scores on this factor were significantly greater in FCD and CCD than in HC. *Gain-Loss* activity in bilateral ventral striatum also correlated inversely with impulsivity-related Factor 2 for FCD subjects, but not CCD or HC subjects. Therefore, contrary to our hypothesis, self-reported impulsivity did not relate to ventral striatal activation in CCD. Also contrary to our hypothesis, we found no difference between groups in risk-taking behavior as assessed by subjects’ Risk-Index scores.

We predicted that, in accordance with the reward deficiency syndrome (RDS) hypothesis, both ventral and dorsal striatal activity would be reduced in both CCD and FCD groups, with CCD showing the greatest reductions in ventral striatal activity. We did not find, however, any significant activation differences between HC and CCD for the *Gain-Loss* contrast in any brain region, including the ventral striatum, in contrast to both the RDS and impulsivity hypotheses. We also found no significant ventral striatal *Gain-Loss* activity differences between HC and FCD. It is important to note that although much research on drug abuse has focused on the ventral striatum due to its suggested involvement in reward processing and responses to abused drugs [6], [55], such efforts have yielded conflicting results in comparing drug users to healthy controls during non-drug reward anticipation and receipt, with some studies showing ventral striatal hypoactivity [11], [12], [13] while others reveal either no differences or ventral striatal hyperactivity in drug users [21], [22], [56]. Here, we present two possible explanations for such conflicting results in these non-drug reward-related studies of substance abuse might include study differences in: 1) subject populations and 2) fMRI tasks.

With regard to differences in subject populations, some of the above-cited studies examined subjects primarily dependent on drugs other than cocaine, including alcohol [12], [13], [22] or marijuana [56]. Clearly, comparison of such studies with fMRI studies of cocaine-dependent individuals, such as our study, presents significant difficulties. Additionally, the cocaine-dependent participants in the studies of Asensio et al. [11] and Jia et al. [21] were recruited from treatment centers and therefore were actively seeking cocaine abstinence, whereas many of our CCD participants were not. Thus, participants in these two prior studies of cocaine-dependence would, in terms of recentness of cocaine use and/or withdrawal, lie somewhere between the CCD and FCD groups in our study. Lastly, etiological heterogeneity in cocaine dependence might be a factor, such that in some individuals, reward-system deficiency might best explain the initial motivation to use cocaine (i.e., the RDS hypothesis), while in other individuals, the initial drive to use cocaine might arise more from risk-taking and/or sensation-seeking personality traits (i.e., the ‘impulsivity hypothesis’). According to this theory, the two groups would show opposite changes in ventral striatal activity in response to non-drug rewards. Studies of cocaine dependence thus far might have included individuals of both etiologies, perhaps leading to seemingly conflicting findings.

With regard to differences in fMRI tasks, the Asensio et al. study involved activation of the reward system with erotic stimuli, and therefore was not only non-drug, but also non-monetary based, whereas the Jia et al. study used a monetary-based paradigm, the Monetary Incentive Delay task (MIDT). Similar to the MIDT, our Domino task involves monetary rewards, but the reward is received only at the end of several games of up to four minutes duration, and only if the game is won. Furthermore, in our study brain activity was measured during domino chip acquisition or disposal during ongoing gameplay, not at the end of the game. Therefore, the gains and losses in the Domino task represent a level of abstraction removed from the already abstract monetary gains and losses in the MIDT, which, in turn, contrasts with fMRI reward-related studies that use “concrete” non-drug rewards, such as food (juice) [57], [58], [59] or sex (erotic stimuli) [11], [60].

Therefore, despite studies reporting deleterious consequences of chronic cocaine use on the mesocorticolimbic reward system [61], [62], [63], and due to noteworthy inconsistencies in findings thus far, it remains unclear if cocaine-dependent individuals in a given study sample will demonstrate ventral striatal hypoactivity or hyperactivity during anticipation or receipt of all types of non-drug rewards (e.g., abstract or concrete rewards), or only under specific circumstances (e.g., during early withdrawal, in the context of continued cocaine abuse, etc.). Our own findings show no differences between CCD and HC individuals, further demonstrating the lack of consistency in findings in cocaine studies examining striatal responses to non-drug rewards. Taken together, these studies indicate that more research is needed to understand the apparent differences in findings across studies and the extent to which specific individual differences might be contributing to these findings.

We can at this stage only speculate about the possible causes for our results showing relative FCD hypoactivity in the right dorsal caudate for the *Gain-Loss* contrast and the inverse correlation of *Gain-Loss* contrast effect size with impulsivity-related Factor 2 in that same region. Our finding of right dorsal caudate hypoactivity in FCD subjects during reward receipt will require replication in future studies, and the interpretation of such results will also necessitate the collection of additional data on treatment strategies (both behavioral and cognitive) used by FCD individuals to maintain abstinence.

We present here, however, three possible explanations for our findings in the right dorsal caudate of FCD individuals that future studies might explore. One such explanation is that prior to cocaine use, FCD compared with CCD subjects, had a different neural ‘makeup’ within the striatum, including dorsal caudate, or other mesocorticolimbic regions, such that even after years of abuse FCD subjects were able to achieve abstinence more easily than CCD. A second possibility is that individual variation in predisposition to cocaine dependence might involve dorsal caudate activity differences that are normalized by cocaine use. Prolonged abstinence then leads to an “unmasking” of some these pre-existing differences in dorsal caudate activity. In other words, once cocaine use achieves a certain chronicity, continued drug use might be necessary to ‘normalize’ the reward system [64]. Therefore, when cocaine use ceases, underlying dorsal caudate deficits, such as those involving reduced dopamine D2-like receptors and elevated dopamine transporters [65], might manifest during receipt of non-drug rewards. A third possible explanation is that FCD subjects, all of whom had abused cocaine long-term (>3 months, average 133 months), might have achieved lasting abstinence through the development of cognitive and behavioral strategies (e.g., group or individual psychotherapy, self-restraint/willpower) necessary to substantially reduce and resist cocaine craving. Such cognitive strategies might have included acquiring the ability to inhibit craving- and habit-related signals arising from the dorsal caudate during reward receipt. This proposed explanation is consistent with recent studies in which substance dependent individuals were able to use cognitive and behavioral training to reduce self-reported cravings and ventral striatal activity when presented with drug cues [66], [67], [68]. It is also consistent with recent studies demonstrating that pharmacological (GABA-receptor agonist) blockade of activity in the dorsal striatum significantly reduced cue-induced cocaine-seeking in rats, suggesting that similar reductions in dorsal striatal activity would reduce cocaine-craving in human cocaine dependence [32], [69].

Therefore, reduced dorsal caudate activity during reward receipt observed in our FCD subjects, rather than representing a dysfunctional response, might instead represent a successful cognitive strategy that allowed these individuals to remain cocaine abstinent by not activating drug-habit-associated brain regions. Our finding that FCD subjects’ scores on self-reported compulsivity and reward-punishment sensitivity correlated negatively with right dorsal caudate activity for the *Gain-Loss* contrast is also consistent with Volkow’s cognitive control hypothesis [66]. The inverse correlation of self-reported compulsivity/reward-punishment sensitivity scores with right dorsal caudate activity might suggest that the most compulsive FCD subjects need to exert the greatest cognitive control to maintain abstinence. Additionally, this inverse correlation indicates that the less compulsive/impulsive FCD subjects tended to mitigate the between-group difference for the *Gain-Loss* contrast in that brain region. CCD subjects scored similarly to FCD on impulsivity-related Factor 2 (and both scored significantly greater than controls), and yet did not show *Gain-Loss* contrast hypoactivity in the dorsal caudate, nor *Gain-Loss* correlation with Factor 2. This result might indicate an unmasking of underlying dysfunction in the FCD group through long-term abstinence.

Although the ideas presented here are at this stage largely speculative, and these results will require replication in future studies, when taken together with findings that impulsivity measures correlate inversely with ventral striatal activation in substance-dependent and pathological gambling populations [12], [13], [36], these concepts resonate with models of ventral-to-dorsal striatal function underlying impulsive-to-compulsive aspects of addictions [26], [29], [31], [70], [71].

Study limitations include reliance on CCD and FCD individuals’ self-reports of durations, amounts and frequencies of cocaine use, and, in the case of the former users, the length of abstinence. CCD and FCD subjects, however, provided at least two urine samples to verify current users were cocaine-positive and former users were not. Another possible limitation is that nine CCD subjects had comorbid opiate abuse and/or dependence treated with stable methadone doses at the time of testing. Re-analysis of CCD subjects’ fMRI data with opiate users removed, however, revealed no changes from results presented in Figure 3. Effect size plots were essentially identical to those depicted in Figure 4, panel B, and peak difference occurred at the same voxel (*x,y,z*  = 18,18,9) in a similarly sized cluster in the right dorsal caudate. Other *past* psychiatric co-morbidity, which were more prevalent in the FCD group, might be confounding our results. However, the fact that removing these participants from the analysis did not have a significant impact on the finding of relative hypoactivity in the right dorsal caudate of FCD group during reward receipt indicates that cocaine use rather than psychiatric co-morbidity is the principal determinant of our results. Also, both current and former cocaine dependent subjects reported varying amounts of weekly cocaine use and lifetime durations of use and differences in age at first use that could potentially impact brain function. Future, larger studies could examine these factors directly.

We should also note the significant difference in weekly spending on cocaine between the FCD and CCD groups. While self-reported weekly spending in the FCD group is considerably higher than the CCD group, we believe that this difference might, at least in part, reflect under-reporting by the CCD group. A study by Harrison and Hughes [72] found that, with respect to recent cocaine users (i.e., the CCD group), “over the course of the 17-week clinical trial, subjects reported cocaine use on 20 percent of occasions, but tested positive for cocaine (qualitatively) on 68 percent of occasions.” Similar studies on self-reported usage in current cocaine abusers have also shown that such users tend to under-report [73], [74], [75]. Therefore, we speculate that many of the CCD individuals in our study may have been under-reporting their current usage. This under-reporting might be due to the desire of CCD individuals to conceal the magnitude of their ongoing illicit drug use from both law enforcement and from healthcare professionals. The FCD group in our study, in contrast, might not have such reservations in self-reporting past cocaine usage. However, we acknowledge that the significant difference in self-reported weekly spending on cocaine between the former and current cocaine groups is a potential confound.

Finally, we should note that we were unable to determine precisely the last use of cocaine by CCD subjects before their fMRI scan. A positive urine test for cocaine generally indicates that the individual had used cocaine within the previous 72 hours [76]. Due to cocaine’s short half-life of 40–60 minutes [77], this implies that some urine-positive CCD subjects in our study might have been in a state of early withdrawal, with symptoms including depressed mood, fatigue, and psychomotor retardation or agitation [78] that might have impacted their reward system response. On the other hand, some urine-positive CCD subjects might have used cocaine comparatively recently prior to scanning (<3–6 hours), so as to have hypothetically ‘normalized’ their reward system at the time of scan. Hence, in CCD participants, variation in amount and recency of cocaine use and state of intoxication/withdrawal at the time of fMRI scan might have added variance or ‘noise’ that obscured underlying differences in reward system function in these subjects versus FCD and HC.

### Conclusions

In summary, during receipts of rewards versus punishments in an interpersonal competitive game involving risk-taking, FCD but not CCD subjects showed altered striatal activation compared with HC subjects. Furthermore, these activation differences were greatest in right dorsal caudate, a region associated with cocaine craving and habit-based behavior such as occurs in drug addiction and, for FCD individuals only, were negatively correlated with an impulsivity-related factor associated with compulsivity and sensitivity to reward and punishment. To our knowledge, our study is one of the first to examine brain reward system function in FCD subjects, *i.e.*, individuals with long term cocaine abstinence (>6 months), thus filling an important gap in the study of cocaine addiction. Future studies should be directed towards determining explanations for the persisting significance of the impulsivity-related factor of self-reported compulsivity and reward-punishment sensitivity in cocaine-dependent individuals, even after prolonged cocaine abstinence. Finally, future research should examine the extent to which dorsal striatal function might represent a target for treatment development in cocaine dependence.
